# Trends in Asthma Morbidity and Mortality in Japan between 1984 and 1996

**DOI:** 10.2188/jea.12.217

**Published:** 2007-11-30

**Authors:** Shinichi Tanihara, Yosikazu Nakamura, Izumi Oki, Toshiyuki Ojima, Hiroshi Yanagawa

**Affiliations:** 1 Department of Environmental Medicine, Shimane Medical University.; 2 Department of Public Health, Jichi Medical School.; 3 Vice-President, Saitama Prefectural University.

**Keywords:** asthma, morbidity, mortality, fatality

## Abstract

Purpose: To determine whether the increased prevalence of asthma in Japan has influenced its mortality. Materials and Methods: A descriptive study was conducted by the data obtained from Patient Survey and Vital Statistics of Japan between 1984 and 1996. Asthma fatalities were expressed as the number of deaths from asthma per 100,000 asthmatic patients receiving medical treatment on the day when the survey was conducted. Results: Mortality, prevalence and asthma fatalities showed different changing patterns among several age groups. Asthma mortality for the 10-24 and 25-44 year-old groups increased during the study period, while for other age groups, it decreased. The prevalence increased for all groups classified by age and sex. Asthma fatalities peaked in 1987 among the 10-24, 25-44 and 45-64 year-old groups, and decreased for others throughout the study period. Conclusions: There is a possibility that an age-specific phenomenon is at work here because asthma mortality increased only in the 10-24 and 25-44 year-old groups, although the prevalence of asthma increased in all groups, whether classified by age or sex. The asthma fatality of the 10-24, 25-44 and 45-64 year-old groups peaked in 1987: it is conceivable that this was influenced by the particular drug therapy used. The increase in asthma mortality in the 10-24 and 25-44 year-olds might be influenced by the increased prevalence.

## INTRODUCTION

An increase in mortality from asthma occurred in the mid-1960s in New Zealand, Australia, England, and Wales^1^^)^. It had increased from the latter half of the 1970s in many countries^2^^-^^7^^)^, especially in the 5-34 year-olds^2^^-^^5^^,^^7^^)^. It was reported that the medications for asthma brought changes in asthmatic mortality^8^^,^^9^^)^. Although the reported prevalence of this disease increased in various countries and age groups^10^^-^^18^^)^, the relationship between asthma morbidity and mortality has not been examined in detail. Only a few studies reported the case-fatality^19^^)^ and mortality/morbidity ratios^20^^)^. In other words, it is still unclear whether the change in mortality was due to the increase in morbidity, a rise in fatalities, or both. The purpose of this study is to determine whether the increase in the prevalence has influenced its mortality in Japan.

## METHODS

Mortality, prevalence, and fatalities in asthma were calculated from data obtained from Patient Survey and Vital Statistics of Japan after 1984, when the current survey methods were adopted.

From Vital Statistics of Japan between 1984 and 1996, age-specific asthmatic mortality was calculated every 3 years. The patients were assigned to age groups: 0-9, 10-24, 25-44, 45-64, 65-74, and 75+ years. During the years specified above, asthma was classified according to the International Classification of Diseases (ICD) 493 (ICD 9th, 1984-1994), and J43 and J44 (ICD 10th, 1995-1996).

To study the trends in prevalence, the total number of patients receiving medical treatment for asthma estimated in Patient Survey was divided by age and sex. The total numbers of patients with the disease, including those out-patients who had not visited a hospital or clinic on the survey day, were obtained, classifying them by age and sex. The total number of asthmatic patients was computed by using the following formula^21^^)^:

$$\begin{array}{rl} & \text{The number of asthmatic patients}\\ & =\text{number of inpatients on the survey day}\\ & +\text{number of out-patients }(\text{those visiting a facility for the first time})\\ & +\text{number of out-patients }(\text{making a return visit on the survey day})\\ & \times \text{average time from the last visit}\times \text{adjusting index }(6/7) \end{array}$$

A fatality has been defined as the death rate observed when a designated series of persons affected by an event^22^^)^. In this study, an asthmatic fatality is expressed as the number of deaths from asthma in one year per 100,000 asthmatic patients receiving medical treatment on the survey day.

## RESULTS

The age- and sex- specific mortalities in Japan are shown in Table 1. For all age groups, the mortality among males was higher than among females during the periods of the study. For both sexes, the mortality of the 75+ group was the highest. Among males in the 10-24 and 25-44 groups it increased slightly while for those in the other age groups, it decreased during the same period. Among females, the mortality for the 10-24 and 25-44 groups increased, starting in 1984. It peaked in 1990. For the other age groups, downward trends were observed as in males.

**Table 1.** The age- and sex- specific mortality rate of asthma in Japan.

(/100,000 population/year)					

Year	1984	1987	1990	1993	1996
Age		Male			
0- 9	0.72	0.52	0.35	0.48	0.30
10-24	0.51	0.86	0.99	1.07	0.85
25-44	0.72	0.98	1.04	1.10	1.16
45-64	3.77	4.31	3.50	3.63	2.81
65-74	26.4	21.6	18.0	16.2	14.6
75+	86.7	64.4	56.0	50.4	43.9

Age		Female			
0- 9	0.50	0.41	0.30	0.35	0.27
10-24	0.31	0.42	0.50	0.48	0.37
25-44	0.49	0.67	0.70	0.64	0.55
45-64	2.68	2.96	2.45	2.22	1.86
65-74	13.5	11.6	10.5	9.66	7.78
75+	32.6	25.5	22.9	22.7	20.5

The age- and sex-specific prevalence of asthma is shown in Table 2. The prevalence in the 0-9 group was the highest of all age groups for every year during the survey period. Except for the 25-44 group, the prevalence among males was higher than for females. The prevalence showed increasing trends in all groups when classified by age and sex. The prevalence for the 0-9 group in 1996 was about twice what it was in 1987.

**Table 2.** The age- and sex- specific prevalence of asthma in Japan.

(/100,000 population )					

Year	1984	1987	1990	1993	1996
Age		Male			
0- 9	1760.2	2224.2	2552.1	3444.1	3520.2
10-24	593.6	646.1	714.9	959.0	846.5
25-44	223.4	233.1	292.8	372.0	429.9
45-64	466.9	472.2	435.4	507.5	597.9
65-74	1085.1	1059.5	1097.2	1105.3	1388.9
75+	1241.3	1135.2	1200.5	1234.6	1595.1

Age		Female			
0- 9	1122.0	1560.7	1633.0	2391.5	2392.5
10-24	383.4	356.2	398.3	576.7	553.7
25-44	226.2	237.6	307.0	449.3	506.5
45-64	448.8	479.5	541.5	563.9	645.4
65-74	736.9	683.4	896.3	922.1	1075.6
75+	701.4	714.9	864.6	757.0	1080.6

**Figure 1.**
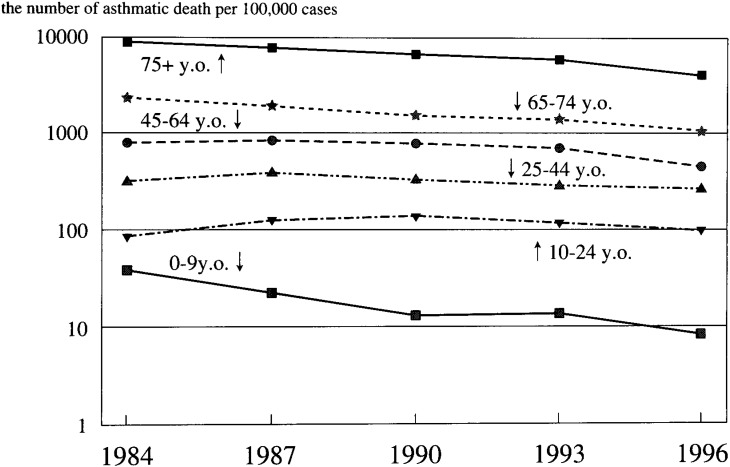
Asthma fatality rates (male).

The asthma fatalities for males are shown in Figure 1. For the 0-9 group, 41 asthmatic deaths per one hundred thousand asthma patients per year were recorded in 1984. Asthmatic fatalities increased as they aged: for the 75+ group, 9,360 deaths per one hundred thousand asthma patients per year were noted.

**Figure 2.**
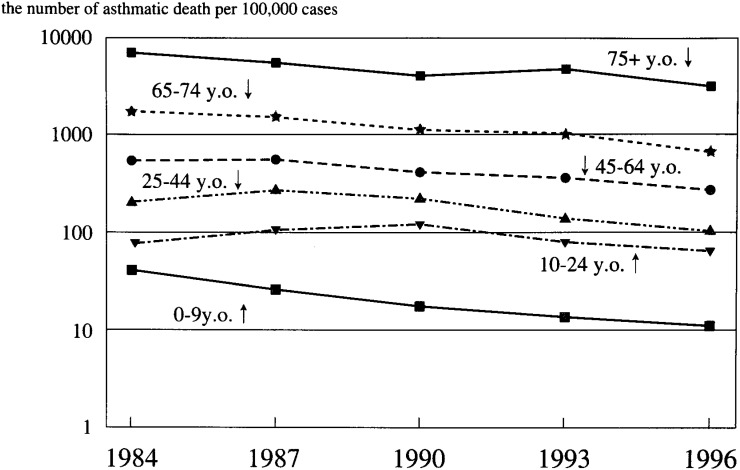
Asthma fatality rates (female).

For the 0-9, 65-74, and 75+ groups, asthma fatalities decreased during the study period. It fell by half in the 65-74 and 75+ groups. For the 0-9 group, a more rapid decrease was observed; and the 1996 fatality was less than one quarter what it was in 1987. For the 10-24 group, asthma fatalities increased by more than 50% between 1984 and 1990 but decreased after 1993. For the 25-44 and 45-64 groups, asthma fatalities peaked in 1987, after which these trends reversed. The gap in the fatalities between the 0-9 and 10-24 groups increased each year, culminating in the former being 12 times the latter in 1996. Asthma fatalities for females are shown in Figure 2. In the 0-9 group, 45 asthma deaths were counted per one hundred thousand asthma patients per year in 1984. Asthmatic fatalities increased by age as in males. For the 75+ group, 7,240 asthmatic deaths were counted per 100,000 asthma patients per year. Except for the 0-9 group, asthmatic fatalities for females were lower than for their male counterparts during the study period.

For the 0-9 and 65-74 groups, asthmatic fatalities decreased continually during the observation periods. Although it was not constant, the rates for the 45-64 and 75+ groups decreased from 1987 to 1996. For the 25-44 group, the rate peaked in 1987, then it began to decrease. For the 10-24 group, the rate increased by more than 50% from 1984 to 1990, then decreased after 1993. Except for the 10-24 group, the rates decreased to less than half for all age groups during the study period. For the 0-9 group, the rate in 1996 was less than a quarter of what it was in 1987. The gap between the 0-9 and 10-24 groups widened each year; however, it was smaller than that for males during the study period.

## DISCUSSION

The major index used in this study, asthma fatality, is defined as the number of deaths from asthma in one year per 100,000 asthmatic patients under medical treatment. It should be noted that “fatality” in this study is defined as that with its numerator determined by the number of asthmatic death for that year. It is impossible to distinguish the factors associated with the increase in fatalities without considering the change in asthma morbidity. Most of the time series analyses of asthma mortality were not concerned about the increase in reported prevalences of asthma^2^^-^^7^^)^. Both increases in morbidity and fatalities may change the mortality for asthma. Only a few studies have reported on the case-fatality^19^^)^ and mortality/morbidity ratio^20^^)^. When discussing the trends in asthma mortality, asthma fatalities are more useful than mortality because they can be used to analyze the change in the asthma mortality, considering the changes in asthma morbidity.

This study shows that: (1) In the 10-24 and 25-44 groups, asthma mortality increased, although it decreased for the other age groups; (2) The prevalence of asthma increased from 1984 to 1996 for each group by sex and age; and (3) Among the 10-24, 25-44 and 45-64 groups, asthma fatalities peaked in 1987, while for the other groups, this statistic decreased continually.

It has been reported that among elderly persons, the true asthma mortalities were overestimated^23^^,^^24^^)^. It is often difficult to distinguish between asthma and other obstructive respiratory diseases with asthma-like symptoms, especially in the very young and the elderly^23^^,^^25^^)^. Death certificates may be inaccurate, the degree of inaccuracy being greater for the elderly,24 children, and young adults^26^^)^. For the 5-45 group, it is generally accepted that the diagnosis of asthma is reasonably reliable^27^^,^^28^^)^. In the current study, asthma mortality for the 10-24 and 25-44 groups increased during the survey period. Similar trends were observed in these age groups in many countries^2^^-^^5^^,^^7^^)^. It can be inferred that the different trends in asthma mortality in these age groups were not simply due to the difference in clinical diagnoses or characteristics of the age.

The change in the codes used to measure mortality may be one of the factors associated with the trends in asthma mortality^29^^)^. In Japan, part of a death certification is classified under ICD 9th and ICD 10th simultaneously^30^^)^. The ratio of the number of death certifications for asthma classified by ICD 9th and ICD 10th was 1.001. A change in codes might not have affected the results too much.

In this study, asthma prevalence was based on the Patient Survey, which is composed of data on patients who visited hospitals and clinics on a day in October. Data on time trends should be assessed with caution because the clinical criteria for diagnosing asthma may have changed with time. Our estimations have been extended to include those who visited medical facilities. Therefore, the number of patients and prevalence were affected by fluctuations of the disease with seasons, the time duration from disease onset through the end of the disease, progress made in diagnostic methods, and changing criteria of the disease. It is believed that fluctuations with the season have very little influence on the results because the Patient Survey was conducted on one day in October during the study period.

It is difficult to estimate the duration of asthma because it is a chronic disease with an insidious onset and ill-defined recovery. There are possible explanations that an increased awareness and improvements in managing this disease^6^^-^^9^^,^^31^^)^ may have caused the time under asthma treatment to be prolonged. This may be one of the causes for the increase in the prevalence of asthma.

The reported prevalence of asthma increased in various countries and age groups^10^^-^^18^^)^. The changes in diagnostic modalities, together with an increased frequency of detection, may have contributed to the upward trends in the prevalence of asthma^4^^,^^10^^)^. In Japan, advances in laboratory test methods may have changed the diagnostic criteria and increased the number of reported cases of allergic rhinitis^32^^)^. However, the observed increase in asthma was not simply due to an increased awareness of asthma, reporting and labeling bias because it has been reported that the prevalence of asthma had increased anyway, even when the same diagnostic criteria were used during the study periods^12^^,^^14^^)^.

Asthma fatalities decreased after 1990 for all age groups in this study. In Canada, mortality/morbidity ratios were observed between 1971 and 1983. The trends showed a peak in the middle of this period^20^^)^. Among hospitalized US veterans, asthma fatalities declined between 1970 and 1987^19^^)^. This may have been due to changes in the severity of asthma^33^^)^, improved treatments, and birth cohort-related changes in smoking exposure^3^^,^^19^^)^.

There is a possibility that risk factors for asthmatic deaths are associated with age because asthma mortality increased only in the 10-24 and 25-44 groups, although the prevalence of asthma increased among all groups when classified by age and sex. It was reported that inhaled fenoterol increases the risk of death in patients with severe asthma in the 5-45 group^34^^)^; and that the sale of specific types of anti-asthma medications was related to the trends in asthma mortality^8^^,^^9^^)^. The observed decrease in asthma fatalities implies that the influence of anti-asthma treatment on asthma mortality is moderated and coincidental with the decrease in mortality and the sale of fenoterol in New Zealand^8^^)^. In Japan, asthma mortality closely paralleled the change in the sale of β_2_-agonist metered dose inhalers during 1950 and 1993^35^^)^. One should be cautious in interpreting the different trends in age-specific asthma mortality. Further research will be required to determine the reasons for these different trends in mortality and fatalities by age.
